# Left atrial myxoma in dextrocardia with situs inversus totalis

**DOI:** 10.1186/1749-8090-10-S1-A104

**Published:** 2015-12-16

**Authors:** Joseph Gabriel, Ishtiaq Ahmed

**Affiliations:** 1 Brighton and Sussex Medical School, University of Sussex, East Sussex, BN1 9PX, UK; 2 Sussex Cardiac Centre, Royal Sussex County Hospital, Brighton, East Sussex, BN2 5BE, UK

## Background/Introduction

We present the rare case of a 69-year-old female, with a history of dextrocardia with situs inversus totalis presenting to her general practitioner with a six-month history of shortness of breath on exertion and pre-syncope. The patient was diagnosed with dextrocardia at age 10, and situs inversus incidentally on total hysterectomy at age 42.

## Aims/Objectives

To report and show video of this rare case of left atrial myxoma in dextrocardia with situs inversus totalis and highlight the diagnostic and ergonomic considerations necessary for the optimal operative management of this condition.

## Method

Echocardiography demonstrated a mass consistent with left atrial myxoma obstructing the mitral valve orifice. CT angiography confirmed true situs inversus with right-sided aortic arch, dextrocardia, left-sided SVC and IVC, right-sided descending thoracic aorta, left-sided liver and right-sided spleen. A 65 × 38 × 34 mm mass arising from inter-atrial septum was visualised extending into the LV.

Coronary anatomy showed no significant lesions.

A trans-septal approach was used, with reconstruction of the inter-atrial septum. The surgeon performed the operation from the contralateral side to facilitate vision and exposure. The septum was initially inadvertently incised too anterior gaining access to the aorta. This was easily repaired and a second more posterior incision gained access into the left atrium.

## Results

Surgery was successful, with post-op TOE showing intact septum and AV-valve. Histopathological analysis confirmed the diagnosis. The patient was transferred to HDU. Fast AF Day 1 post-surgery was treated successfully with DCCV and she was discharged on day 10. At 2 month follow-up, she was well and asymptomatic.

## Discussion/Conclusion

Operating on left atrial myxoma in dextrocardia with situs inversus presents a unique operative challenge, requiring briefing the entire operating team of the planned approach, and remaining mindful of the ergonomics of theatre. Cardiac theatre by nature is highly consistent in the set-up for routine cases. In such a case, positioning of the surgeon, assistants and perfusionist is altered. Usual anatomical landmarks and topography are distorted, facilitating inadvertent entry into the incorrect chamber. Good planning and briefing are essential for optimal technique and patient outcomes.

## Consent

Written informed consent was obtained from the patient for publication of this abstract and any accompanying images. A copy of the written consent is available for review by the Editor of this journal.

